# Passing through Open/Closed Doors: A Solution for 3D Scanning Robots

**DOI:** 10.3390/s19214740

**Published:** 2019-10-31

**Authors:** Samuel A. Prieto, Antonio Adán, Andrés S. Vázquez, Blanca Quintana

**Affiliations:** 3D Visual Computing and Robotics Lab, University of Castilla-La Mancha, 13005 Ciudad Real, Spain; Antonio.Adan@uclm.es (A.A.); Andress.Vazquez@uclm.es (A.S.V.); Blanca.Quintana@uclm.es (B.Q.)

**Keywords:** robotic platform, robot interaction, 3D data processing

## Abstract

In this article, a traversing door methodology for building scanning mobile platforms is proposed. The problem of passing through open/closed doors entails several actions that can be implemented by processing 3D information provided by dense 3D laser scanners. Our robotized platform, denominated as MoPAD (Mobile Platform for Autonomous Digitization), has been designed to collect dense 3D data and generate basic architectural models of the interiors of buildings. Moreover, the system identifies the doors of the room, recognises their respective states (open, closed or semi-closed) and completes the aforementioned 3D model, which is later integrated into the robot global planning system. This document is mainly focused on describing how the robot navigates towards the exit door and passes to a contiguous room. The steps of approaching, door-handle recognition/positioning and handle–robot arm interaction (in the case of a closed door) are shown in detail. This approach has been tested using our MoPAD platform on the floors of buildings composed of several rooms in the case of open doors. For closed doors, the solution has been formulated, modeled and successfully tested in the Gazebo robot simulation tool by using a 4DOF robot arm on board MoPAD. The excellent results yielded in both cases lead us to believe that our solution could be implemented/adapted to other platforms and robot arms.

## 1. Introduction

Autonomous mobile robots confront different situations when navigating the interiors of buildings. For example, their paths are frequently blocked by unexpected obstacles and they must avoid collisions by using their own local planning algorithms. However, when a robot has to pass from one room to another, several problems must be solved, including a possible interaction with the door handle.

Robots that must open doors are, for example, robotic wheelchairs that help handicapped people to move in different environments [1,2,3]. Other assistive robots open closets or cupboard doors, or help humans with their daily routines at home [4]. Rescue robots also require the ability to open doors.

In this work, we deal with the problem confronted by autonomous scanning mobile platforms when they have to traverse closed doors in buildings. To the best of our knowledge, no similar data on scanning robots in that particular framework have been published to date.

The automatic opening and traversal of doors is a robotic problem that has been studied for more than 25 years. One of the earliest works is that of Nagatani et al. [5], who defined some strategies for a simulated mobile manipulator. The same authors later implemented these strategies in a real robot [6]. One of the most recent exciting forums on the opening and traversing of doors was developed in one of the trials in the 2015 DARPA robotics challenge [7,8], while even more recently, the impressive SpotMini robot was presented by Boston Dynamics in [9].

Opening doors can involve a large number of processes that robots must perform. The majority of works published to date have focused on presenting a compilation of techniques, ranging from door/handle detection to the generation and control of robot trajectories. We have employed a point of view based on the type of the sensor used to establish a distinction between techniques based on force or vision sensors.

In the first case, the force/torque sensors are usually installed in the wrist of the manipulator or in the end-effector. For example, in [10] the authors present a mobile manipulator that includes a parallel finger with force sensors attached to the tips of its fingers. Several solutions regarding the handle pose or the detection of locked doors are proposed. These kinds of sensors are generally used to overcome the problem of uncertainty in terms of positioning, mechanical issues and external forces in doors. One of the research lines, which has been developed at the Centre for Autonomous Systems (KTH), is focused on opening doors in situations of this kind of uncertainty. The aforementioned research centre has produced a predictive controller that is able to model unknown doors, which is presented in [11]. This controller calculates the centre of rotation and radius of the handle by using the forces measured in the wrist of the robot when the door is opened. A controlled trajectory is also generated according to this model. More recently, the authors have proposed a model-free adaptive velocity-force/torque controller for simultaneous compliant interaction and the estimation of doors and drawers [12]. An interesting and complete review of previous door opening works can also be found in the paper in question.

Although it is obvious that force/torque controllers can be very useful for door opening and traversal, they cannot be used in some environments and applications, such as rescue missions during nuclear disasters [8,13], military missions and law-enforcement actions [14], since the force/torque sensors do not have the required durability.

With regard to vision-based techniques, a combination of 2D and 3D vision sensors are frequently used in order to obtain a simple map of the environment in which the robot safely navigates, or a semantically rich 3D model of the scene [15], which contains richer information. The Willow Garage’s PR2 robot [16] uses a 2D laser sensor for robot navigation and a tilting laser (3D sensor) to obtain the model of the scene, including doors and handles. In [17], the authors use a Microsoft Kinect (3D sensor) to recognize doors and handles, while a 2D laser scan is used to estimate the position and orientation of the door. In the aforementioned 2015 DARPA Challenge, the Atlas robot (Boston Dynamics [8]) detected doors by using both 2D and 3D data originating from a Hokuyo LIDAR and a stereo camera, respectively. Andreopoulos et al. [2] propose a robotic wheelchair, carrying a 2D camera, that recognizes doors and handles. In order to reduce the uncertainty, 2D/3D sensors have also been used together with other kinds of sensors. For example, Axelrod et al. [14] place a tactile sensor on the fingers of the robot’s gripper to ensure that it is correctly aligned with the handle.

In our approach, we use two 2D laser sensors for robot positioning purposes and a 3D laser scanner (Riegl VZ-400) to recognize the door and the handle. The main difference between this and other works lies in the fact that our approach is based on the 3D model previously obtained. This semantic model contains detailed information concerning the geometry of the interior of the building (including doors), which is then used to open and traverse them.

With regard to the issue of trajectory planning, some proposals solve the problem by using static or quasi-static methods, while others deal with the dynamics of the robot–door-handle set.

Static/Quasi-static methods are implemented on robots without force/torque sensors and are less robust than the dynamic ones. However, many works have provided feasible solutions. For example, in [18], Li et al. present a path planning algorithm based on inverse kinematic solutions and Jacobians. The authors prove that the manipulator joints exert sufficient torque to twist the handle and open the door during the trajectory. Our method can also be classified as a static method. The difference with respect to other static methods lies in the fact that we solve the path planning problem by employing the information contained in our 3D semantic model and, particularly, in a dense point cloud of the door.

In contrast, dynamic methods deal with the forces produced during the opening action and, therefore, use force/torque sensors. One example of a dynamic method is that of Chen et al. [19], in which an adaptive trajectory that suppresses modelling errors, joint frictions and external disturbances, is proposed.

This paper focuses on showing a solution to the issue of enabling a mobile scanning platform to pass through doors. Section 2 is devoted to presenting an overview of our scanning system and identifying the stages regarding the traversal door problem. Section 3 and Section 4 provide a short explanation of the first stages of the process: moving towards the exit door and handle recognition. Section 5 presents a detailed formulation of the different steps in the general stage of opening closed doors. Experimentation and results as regards representative case studies are addressed in Section 6, and finally, Section 7 presents our conclusions and future work.

## 2. Overview of the Method

This section presents an overview of our approach for the navigation of mobile robots through closed doors. As will be seen in the following subsections, our method has been adapted to our Mobile Platform for Autonomous Digitization (MoPAD) but is easily generalizable to similar mobile platforms.

**Figure 1:**
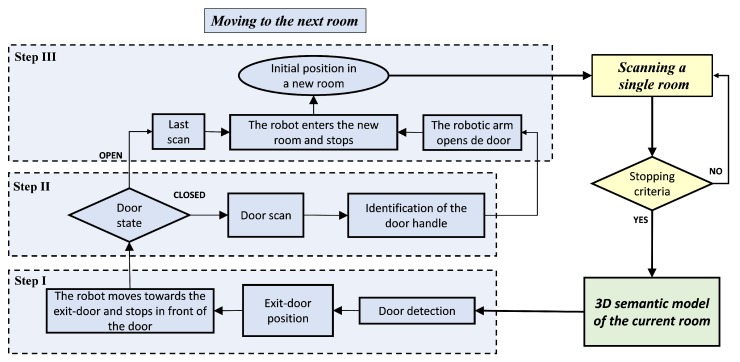
Main modules of the whole scanning process: data acquisition (yellow), 3D modelling (green) and moving Mobile Platform for Autonomous Digitization (MoPAD) to a contiguous room (blue).

Figure 1 shows a flowchart that synthesises the whole scanning process. Three colours are assigned in order to distinguish between the main modules of the approach: data acquisition (yellow), 3D modelling (green) and moving MoPAD to a contiguous room (blue). As mentioned in Section 1, this paper specifically deals with the last module. Complete information on the first and second modules can be found in references [20,21]. It is necessary to follow three consecutive steps to complete the action “moving MoPAD to the next room”. In step I (see Section 3), MoPAD stops scanning, identifies the exit-door, recognizes the state of the door, moves towards the exit-door and places itself in front of it. In step II (see Section 4), and if the door is closed, the handle of the door is recognized and positioned by processing a new selective and dense scan of the door. In the third step (see Section 5), MoPAD opens the door and passes through it to the next room. In the case of an open door, steps II and III are simplified, and MoPAD merely has to pass through the doorframe.

As mentioned previously, MoPAD is a mobile robot equipped with a Riegl VZ-400 3D laser scanner and two LiDAR URG-04LX-UG01, one on the back of the robot and one at the height of the Riegl VZ-400. The LiDAR in the rear part is used by the local planner to avoid obstacles that can appear behind the robot.

**Figure 2:**
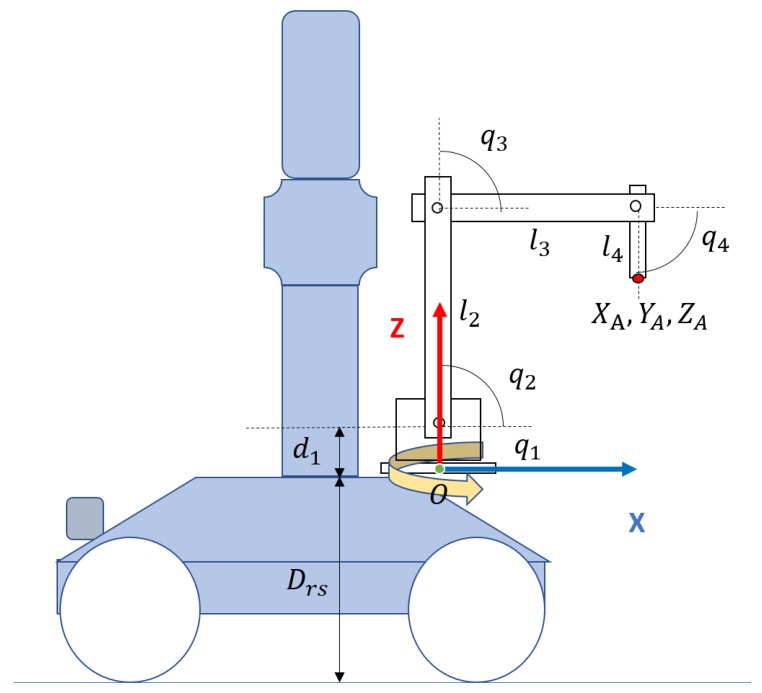
Representation of MoPAD and the 4DOF manipulator on the top of MoPAD.

**Figure 3:**
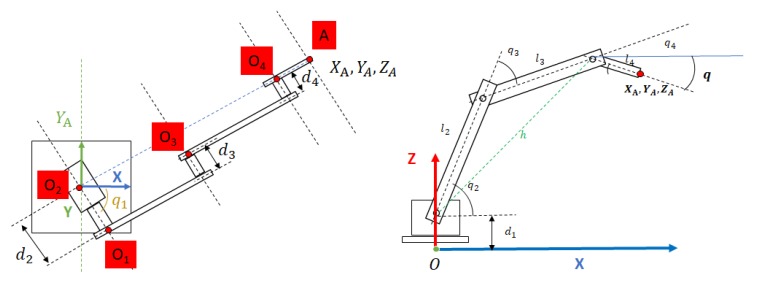
Representation of the reference frames and the joint coordinates used in the kinematics calculation of the robot.

The scanning and semantic modules shown in Figure 1 have been validated through experimentation in real scenarios as presented in references [20,21]. Door and handle recognition modules have been tested in several real scenarios. In Section 3.1 and Section 4 we present, along with an explanation of those modules, some real scene results. In Section 4, we also present the validation of the handle recognition module performed with a test bench of 22 different door handles. The door opening and traversal modules have been validated via experimental simulation using Gazebo [22] (See Section 6.2). In particular, we have designed and simulated a manipulator arm with 4DOF, which is attached to the top of the mobile robot (Figure 2), at a distance $D_{rs}$ from the ground. The Denavit–Hartenberg parameters can be found in Table 1, and correspond with the frames represented in Figure 3. The experimentation with a real robotic arm will be addressed in future work since this robot has not yet been integrated in MoPAD.

This simulation has principally served to verify the validity of our method, along with its performance with a simple manipulator. The use of more complex manipulators, such as colaborative manipulators, would obviously always improve the robustness of the system.

## 3. Step I. Moving towards the Exit Door

### 3.1. Door Recognition and Positioning

MoPAD is designed to collect 3D information (i.e., point clouds) and obtain a simplified 3D model of the scene, which includes structural elements (SE) such as walls, floors, ceilings and columns. The 3D scanner on board MoPAD takes several samples from different positions of the room according to the output of an original next best view algorithm [20], and eventually generates a complete point cloud *S*. The earlier scanning process ends when two criteria are satisfied. The first imposes a high percentage of the scanned SEs area (above 90%), whereas the second requires a minimum gain of the SE area for the last two scans (over 1%).

In order to extract a geometric 3D model of the room, the accumulated point cloud *S* is processed. *S* is first split into vertical and horizontal segments, which represent the existing walls, floor and ceiling. Finally, a coloured-depth image, denoted as a 4D orthoimage $J_{CD}$, is obtained from each of the segments calculated. In the case of a wall-segment, each pixel in the orthoimage $J_{CD}$ therefore contains colour and depth information, which is the orthonormal distance from the point to the fitted wall plane. The existing openings on $J_{CD}$ are subsequently calculated.

Since the door detection algorithm has been published in detail in [23], only a brief explanation is provided here. The discontinuities as regards the colour and depth of the wall are first detected separately—by using the gradient operator for $J_{C}$ and the Canny edge detector for $J_{D}$—and eventually combined into a unique discontinuity image $J_{CD}^{\prime }$. Horizontal and vertical straight lines are then detected in $J_{CD}^{\prime }$. Since it is assumed that these lines might represent door frames, we calculate all possible rectangles defined by two pairs of horizontal and vertical lines, and select only those rectangles whose size falls within the range of typical opening sizes.

The opening angle of the door leaf, λ, is obtained by means of the depth information of the pixels within the previously calculated door frame. This angle is formally defined as the angle of the plane fitted to the interior points and the plane of the wall.

**Figure 4:**
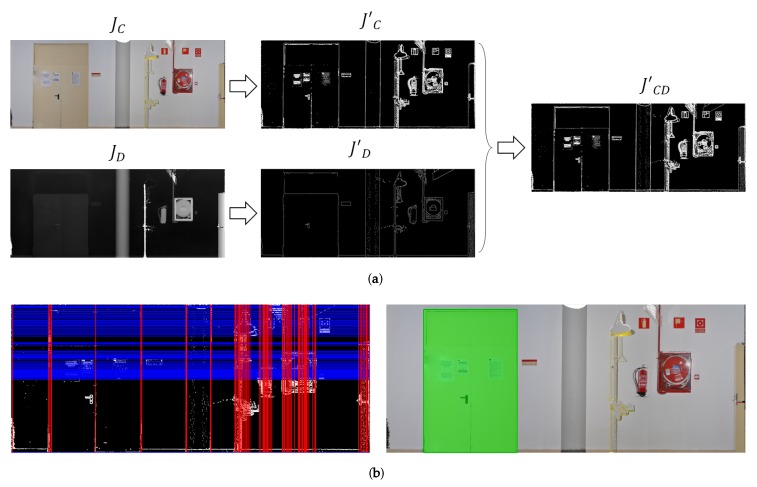
(**a**) Orthoimage $J_{CD}$ separated into colour and depth components, and the combined discontinuity image $J_{CD}^{\prime }$. (**b**) Horizontal and vertical lines detected and the recognized door (highlighted in green).

In conclusion, the outputs are the 3D coordinates of the four vertices $v_{1},v_{2},v_{3}$ and $v_{4}$ (in the 3D model reference system), and the opening angle λ. Figure 4 shows an example of orthoimage $J_{CD}$, the unified discontinuity image, the set of horizontal and vertical lines and the door detected.

### 3.2. Robot Navigation and Final Placement

The autonomous navigation of the MoPAD platform is based on an obstacle map obtained from the point cloud *S*. More specifically, the obstacle map corresponds to the top projection of all the points belonging to *S* that lie below the height of the 3D sensor. While this obstacle map is used by the path planning algorithm, another binary map, obtained from a thin slice at the height of the LiDAR sensor, is used for localization. This enables MoPAD to match the readings of the LiDAR with this localization map, and obtain its current localization by means of an adaptive Monte-Carlo localization algorithm (AMCL). A detailed description of the autonomous navigation process can be found in [21].

**Figure 5:**
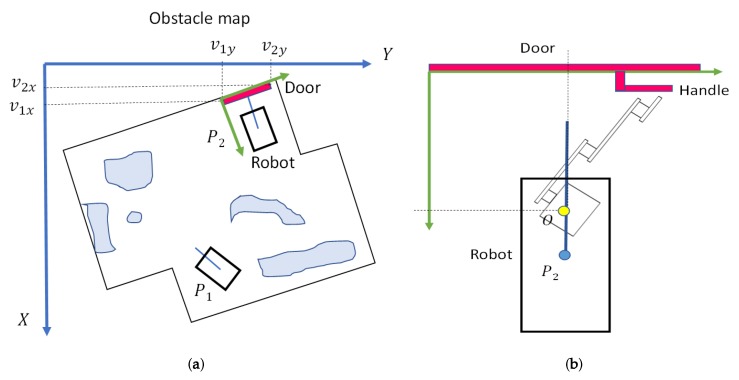
(**a**) Representation on the world coordinate system. Obstacle map and initial and final positions of the robot. (**b**) Position and orientation of the robot in front of the door.

The coordinates of the exit door are then translated to the coordinate system of the obstacle map (see Figure 5a). As it now has the current position of the platform, $P_{1}$, and the coordinates of the exit door, the path planning algorithm can now compute a safe trajectory to a position in front of the door, $P_{2}$. This position is located 150 cm away from the door, with MoPAD oriented perpendicularly to the door’s plane (see Figure 5b). It is in this position that the platform first takes a dense scan of the door and then recognises the door handle.

## 4. Step II. Handle Recognition and Positioning

**Figure 6:**
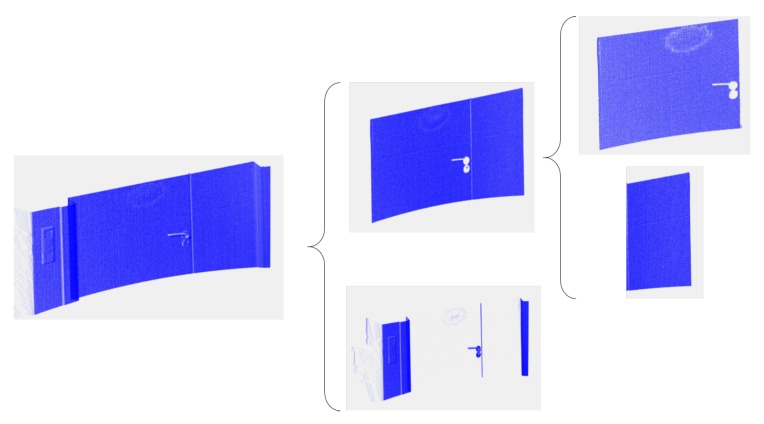
From left to right: the slice of the point cloud taken in front of the door, point clouds fitted to planes $\pi_{1}$ and $\pi_{2}$ and the point clouds of the moving and unmoving parts.

**Figure 7:**
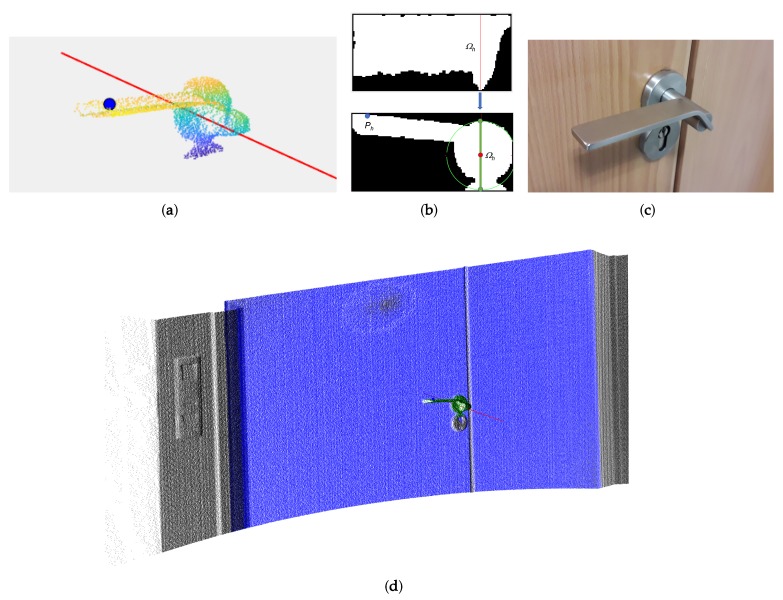
(**a**) Points corresponding to the door handle ($S_{h}$). The handle rotation axis and the contact point are superimposed onto $S_{h}$. (**b**) (Top) Top projection of $S_{h}$ and handle rotation axis in red. (Bottom) Frontal projection of $S_{h}$ and rotation axis (red dot). (**c**) Picture of the door handle. (**d**) Fully segmented point cloud.

The 3D scan taken in front of the door provides a dense point cloud that is later processed in order to recognize the type of handle and its 3D position in the world coordinate system. Four stages have been considered in this process:

- Stage I: Calculation of the door’s plane and its associated points. First, the point cloud is segmented into two parts: one segment corresponds to the points belonging to the door and the other contains the remaining points. The segmentation is carried out by using the MLSAC method, which provides two planes, $\Pi_{1}$ and $\Pi_{2}$, corresponding to both segments.
- Stage II: Defining the moving door leaf. Since the door can be composed of two parts (see Figure 6), which we denominate as moving and unmoving parts, it is necessary to delimitate the moving part of the door. A simple 3D growing region algorithm yields several segments of points, with the largest corresponding to the moving door leaf.
- Stage III: Pulling or pushing door. The type (pulling or pushing) of the door is determined by analysing the relative position of the aforementioned planes $\Pi_{1}$ and $\Pi_{2}$. As is usual in doors, if $\Pi_{1}$ is behind $\Pi_{2}$, the door is pushed, and otherwise it is pulled.
- Stage IV: Door handle and contact point. The protruding points within the door leaf boundaries are assumed to be the points corresponding to the door handle, which we denotate as $S_{h}$. The rotation axis of the door is established as the furthest vertical door edge with respect to the handle.
  In order to identify the rotation axis of the handle, $\Omega_{h}$, and the contact point of the robot arm, $P_{h}$, we process the top and frontal projected images of $S_{h}$ as follows.
  - The top projection of the rotation axis is found as the largest vertical profile in the top projected image, which covers the rod that joins the handle to the door frame. This axis can be appreciated in Figure 7b, Top. Note the noisy points generated by the 3D scanner in the upper part of the figure. This axis has also been used in the frontal projected image in Figure 7b, Bottom, to obtain a perpendicular line in the image. The intersection of this line with the highest and lowest points of the handle defines a segment whose middle point establishes the perpendicular projection of the rotation axis.
  - The contact point, $P_{h}$, is located at the point furthest away from the axis of rotation, with an offset of 10% of the handle length.

**Figure 8:**
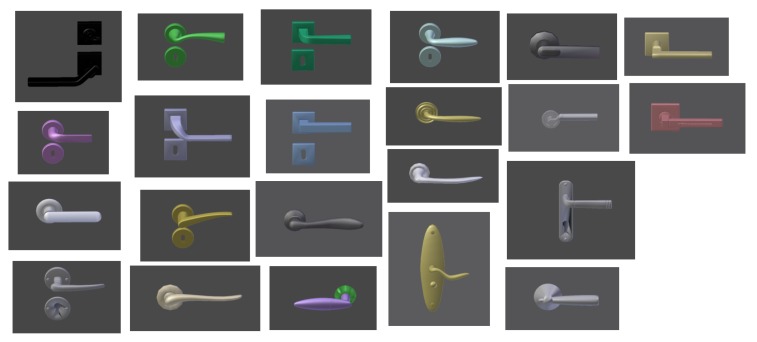
Data base of door handle models for which the approach has been successfully tested.

**Figure 9:**
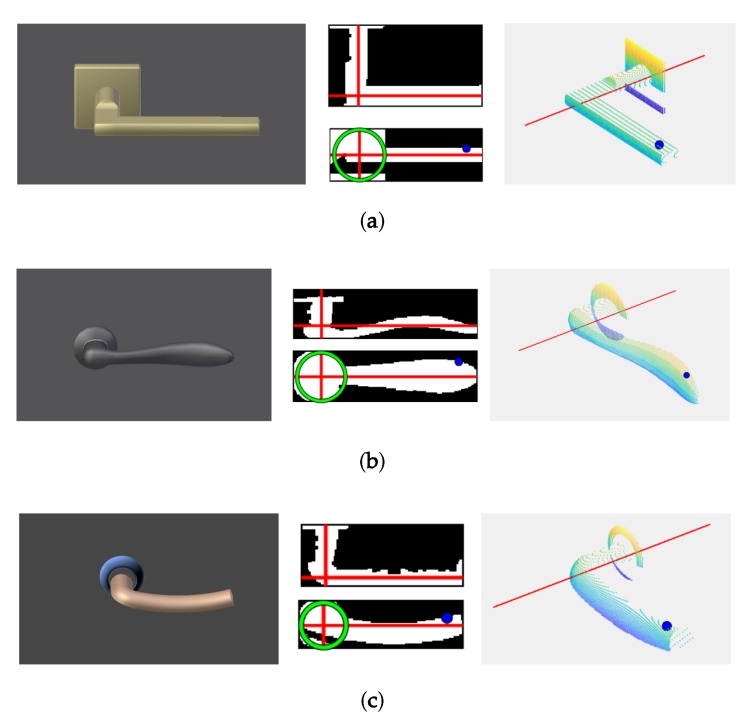

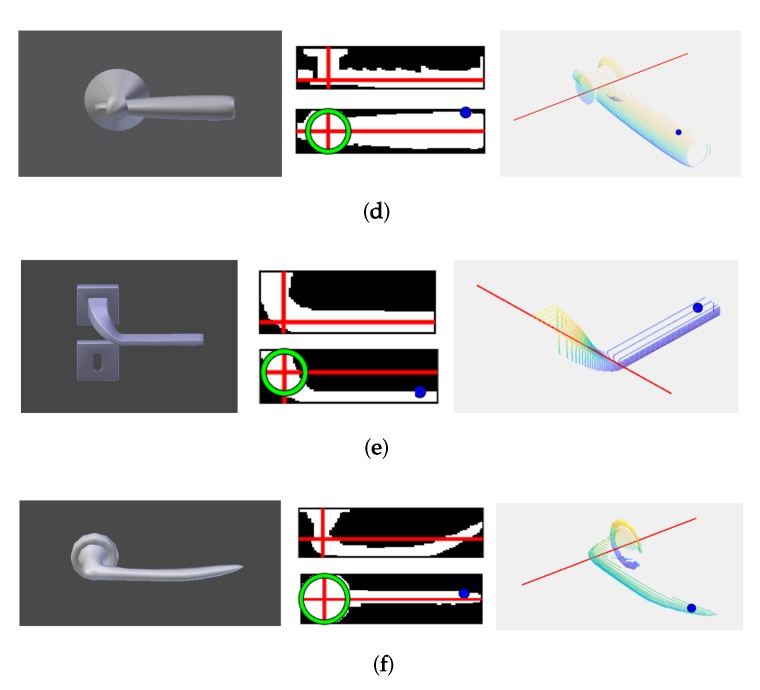
(**a**)–(**f**) Some of the most representative cases of the simulated door handles (left), with both top and frontal projections (middle) and the final point cloud with the axis of rotation Ω_*h*_ and contact point *P_h_* (right).

In order to demonstrate the robustness of this algorithm, extended experimentation was conducted on 22 different types of door handles (Figure 8). The algorithm successfully detected the rotation axis and the contact point in all cases. Figure 9 shows the results for some of the most representative cases.

## 5. Step III. Opening Closed Doors

**Figure 10:**
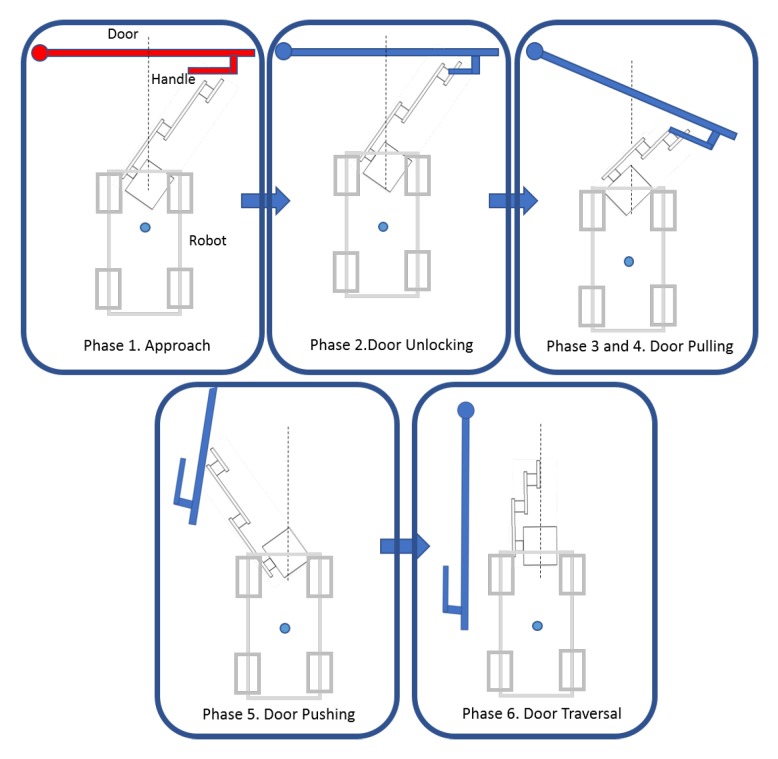
Substeps for opening closed doors.

**Figure 11:**
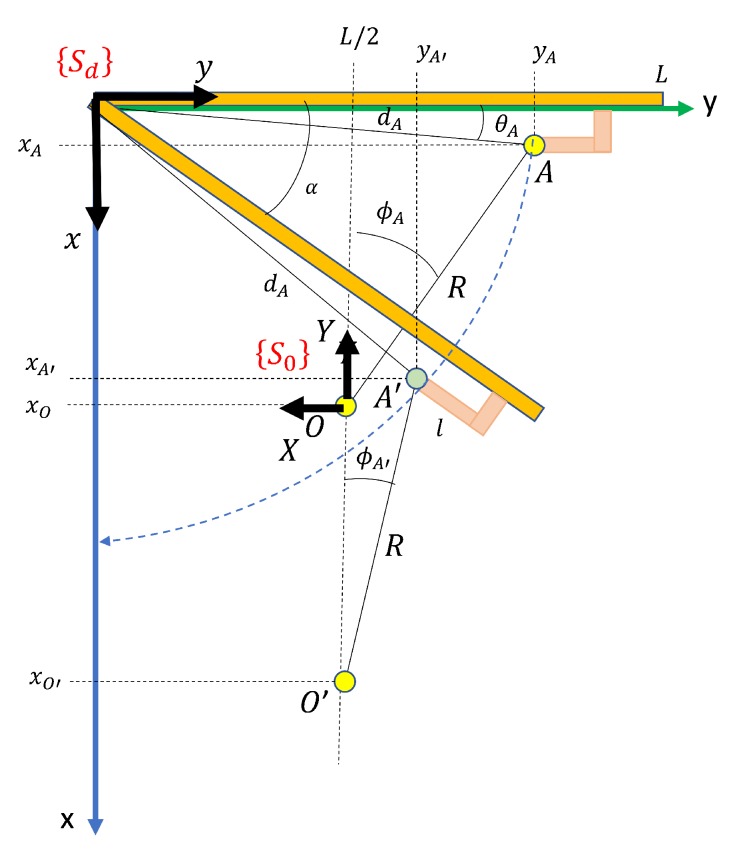
Pulling the door. Reference frames and parameters in initial and intermediate states.

Opening closed doors is the most complex part of the whole process. It begins by pushing down the door handle in order to release the door leaf from the frame, and it ends when the door has been opened sufficiently for the robot to traverse it. The principal steps in this process are illustrated in Figure 10. Figure 11 shows the door reference frame $\left\{S_{d}\right\}$ and the robot reference frame $\left\{S_{O}\right\}$ for initial and intermediate states. The following subsections present the robot arm kinematic formulation for each step. Dynamic control of the mobile robot and the robot arm are not within the scope of this paper.

Let us assume a door of length *L* that is initially closed, which has a handle of length *l* with a contact point *A* of coordinates $\left(x_{A},y_{A}\right)$ with respect to the door frame $\left\{S_{d}\right\}$. As is shown in Section 3, our system processes the 3D point cloud taken by the scanner in front of the door and calculates coordinates $\left(x_{A},y_{A}\right)$ in the frame $S_{P}$. The polar coordinates of *A* are:

(1) $$\begin{array}{c} d_{A}=\sqrt{\left(x_{A}^{2}+y_{A}^{2}\right)} \end{array}$$

(2) $$\begin{array}{c} \theta_{A}=arctan\left(\frac{x_{A}}{y_{A}}\right). \end{array}$$

### 5.1. Phase 1. Approach

In this phase, the mobile robot is placed in front of the door with door coordinates $O(x_{O},y_{O},z_{O})$, where $x_{O}$ is a constant value in this phase, $y_{O}=L/2$ and $z_{O}=D_{rs}$. The end-effector of the robot is initially placed at a vertical distance *h* above point *A* and then moved along the *Z* axis until it reaches the height of the handle. The equations of the end-effector in the frame $S_{O}$ (at this moment) are, therefore:

(3) $$\begin{array}{c} X_{1}=X_{A} \end{array}$$

(4) $$\begin{array}{c} Y_{1}=Y_{A} \end{array}$$

(5) $$\begin{array}{c} Z_{1}=Z_{A}+h. \end{array}$$

### 5.2. Phase 2. Unlocking the Door

Let $\left(x_{c},y_{c},z_{c}\right)$ be the coordinates in $\left\{S_{d}\right\}$ of the centre of rotation of the handle of length *l*

(6) $$\begin{array}{c} x_{c}=x_{A} \end{array}$$

(7) $$\begin{array}{c} y_{c}=y_{A}+l \end{array}$$

(8) $$\begin{array}{c} z_{c}=z_{A}. \end{array}$$

**Figure 12:**
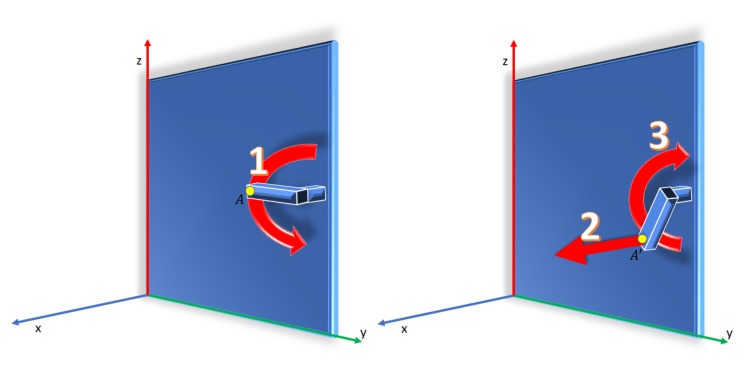
Movement of the end-effector in phase 2. (Left) The bolt is unlocked (1). (Right) The robot arm releases the bolt (2) and the handle returns to its initial position (3).

The phase for unlocking the door consists of two steps (see Figure 12):

- Unlocking the bolt. The end-effector, which comes into contact with *A*, will follow a circular path to release the bolt. Upon imposing an angular speed of the handle $w_{m}$, the coordinates of the moving point *A* in the frame $\left\{S_{O}\right\}$ are:
  (9) $$\begin{array}{c} X_{21}=X_{A} \end{array}$$
  (10) $$\begin{array}{c} Y_{21}=y_{c}-l\cos\left(w_{m}t\right) \end{array}$$
  (11) $$\begin{array}{c} Z_{21}=z_{c}-l\sin\left(w_{m}t\right). \end{array}$$
  The movement is performed until $w_{m}t$ exceeds a preset angle μ (usually $\mu =45^{\circ })$. The time of the movement is $T=\mu /w_{m}$.
- Releasing the bolt. In this step, the end effector moves back ∆X to release the bolt and performs the reverse path reaching the initial position. Finally, the YZ components would be approximately the initials of point *A*. The equations of the end effector coordinates are:
  (12) $$\begin{array}{c} X_{22}=X_{A}-∆X_{A} \end{array}$$
  (13) $$\begin{array}{c} Y_{22}=y_{c}-l\cos\left(w_{m}\left(T-t\right)\right) \end{array}$$
  (14) $$\begin{array}{c} Z_{22}=z_{c}-l\sin\left(w_{m}\left(T-t\right)\right). \end{array}$$

### 5.3. Phase 3. Door Pulling

Let us assume that the door must be pulled. The mobile robot, located at $O(x_{O},y_{O})$, pulls the door by fixing the end-effector to point *A*. Note that the distance *R* between *O* and *A* remains constant while the robot moves back in the *x* direction of the frame $\left\{S_{d}\right\}$. After a time *t*, the robot is now in $O^{\prime }\left(x_{O}^{\prime },y_{O}^{\prime }\right)$ and the grip point becomes $A^{\prime }\left(x_{A}^{\prime },y_{A}^{\prime }\right)$. Since *R* is constant, it can be deduced that:

(15) $$\begin{array}{c} x_{A}^{\prime }=d_{A}\sin\left(wt+\theta_{A}\right) \end{array}$$

(16) $$\begin{array}{c} y_{A}^{\prime }=d_{A}\cos\left(wt+\theta_{A}\right), \end{array}$$

where *w* is the speed of rotation of the door, which is related to the speed of the robot $v_{r}$ (which is assumed to be given). Since ${\hat{AA}}^{\prime }={\hat{OO}}^{\prime }$, we have that $v_{A}=v_{r}=d_{A}w$. Thus:

(17) $$\begin{array}{c} w=\frac{v_{r}}{d_{A}}. \end{array}$$

The coordinates of $O^{\prime }$ are calculated by taking into account that:

(18) $$\begin{array}{c} R^{2}={\left(x_{O}^{\prime }-x_{A}^{\prime }\right)}^{2}+{\left(y_{O}^{\prime }-y_{A}^{\prime }\right)}^{2}, \end{array}$$

from which:

(19) $$\begin{array}{c} x_{O}^{\prime }=\sqrt{R^{2}-{\left(y_{O}^{\prime }-y_{A}^{\prime }\right)}^{2}+x_{A}^{\prime }}. \end{array}$$

Since $y_{O}^{\prime }=\frac{L}{2}$, we eventually obtain:

(20) $$\begin{array}{c} x_{O}^{\prime }=\sqrt{R^{2}-{\left(\frac{L}{2}-d_{A}cos\left(wt+\theta_{A}\right)\right)}^{2}+d_{A}sin\left(wt+\theta_{A}\right)}, \end{array}$$

and the angle $\Phi_{A}^{\prime }\left(\hat{O^{\prime }O,O^{\prime }A^{\prime }}\right)$ is calculated as:

(21) $$\begin{array}{c} \Phi_{A}^{\prime }=arctan\left(\frac{x_{A}^{\prime }-x_{O}^{\prime }}{y_{A}^{\prime }-y_{O}^{\prime }}\right), \end{array}$$

which is the first manipulator joint, $q_{1}$.

The coordinates of the end-effector with respect to the robot system $S_{O}$ are:

(22) $$\begin{array}{c} X_{3}=x_{O}^{\prime }-x_{A}^{\prime }, \end{array}$$

(23) $$\begin{array}{c} Y_{3}=-\left(y_{A^{\prime }}-\frac{L}{2}\right), \end{array}$$

(24) $$\begin{array}{c} Z_{3}=D. \end{array}$$

Note that coordinate $Z_{3}$ remains constant and can be calculated by using the height of the handle with respect to the ground $D_{ms}$ (which was previously calculated from the point cloud of the scene) and the height of the robot base frame $S_{O}$, $D_{rs}$. Formally:

(25) $$\begin{array}{c} D=D_{ms}-D_{rs}. \end{array}$$

**Figure 13:**
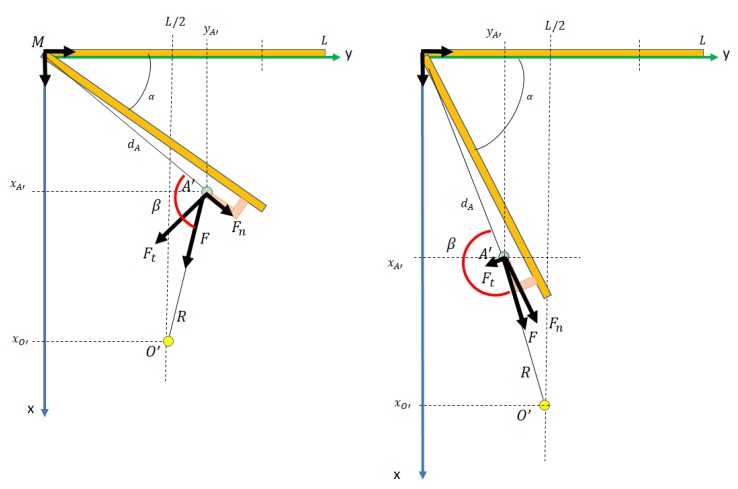
Illustration of forces undergone by the handle during the pulling process. Two moments are shown. In the left-hand picture, the tangential force is sufficiently great to make the door rotate. In the right-hand picture, the tangential force is small and the door will probably not rotate.

In conclusion, the robot moves back on the axis *X* according Equation (20) and turns around axis *Z* according to Equation (21), causing the door to rotate. However, since the force on the tangential component of the path of $A^{\prime }$ decreases as it rotates, the traction movement also decreases. When $\beta (\hat{\vec{A^{\prime }O},\vec{A^{\prime }M^{\prime }}})=\pi$, the tangential component will be zero. A new type of interaction is, therefore, necessary before this occurs. We establish a threshold of $\beta =\frac{2\pi }{3}$. At this moment, the process goes on to phase 4. See Figure 13 for a better understanding of this.

### 5.4. Phase 4. Handle Release and Trajectory to Door Edge

**Figure 14:**
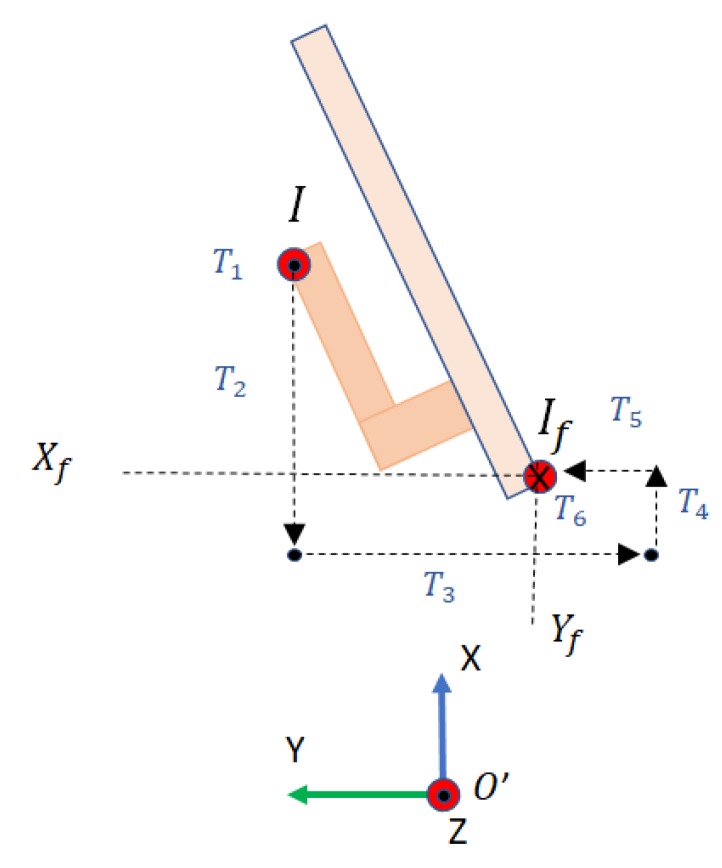
Illustration of the movement of the end effector in phase 4.

In this phase, the handle is released and the end-effector is positioned at the edge of the door at a new contact point $I_{f}$ (see Figure 14). The movement of the end effector is composed of:

- Forward motion on Z. The *Z* coordinate is increased until Z=D+∆Z, where ∆Z is a safety margin. In our case ∆Z=3 cm.
- Backward motion on X. The *X* coordinate is decreased until $X<X_{If}-∆X$, where ∆X is a safety margin. In our case ∆X=3 cm.
- Backward on Y. The *Y* coordinate is decreased until $Y<Y_{If}-∆Y$, where ∆Y is a safety margin. In our case ∆Y=3 cm.
- Forward on X. The original coordinates in *Z* and *Y* are maintained, and the *X* coordinate is increased until $X=X_{If}$.
- Forward on Y. The original coordinates in *Z* and *X* are maintained, and the *Y* coordinate is increased until $Y=Y_{If}$.
- Backward on Z. The original coordinates in *X* and *Y* are maintained, and the *Z* coordinate decreases until Z=D

The final position is

(26) $$\begin{array}{c} X_{4}=X_{If}, \end{array}$$

(27) $$\begin{array}{c} Y_{4}=Y_{If}, \end{array}$$

(28) $$\begin{array}{c} Z_{4}=D. \end{array}$$

### 5.5. Phase 5. Door Pushing

**Figure 15:**
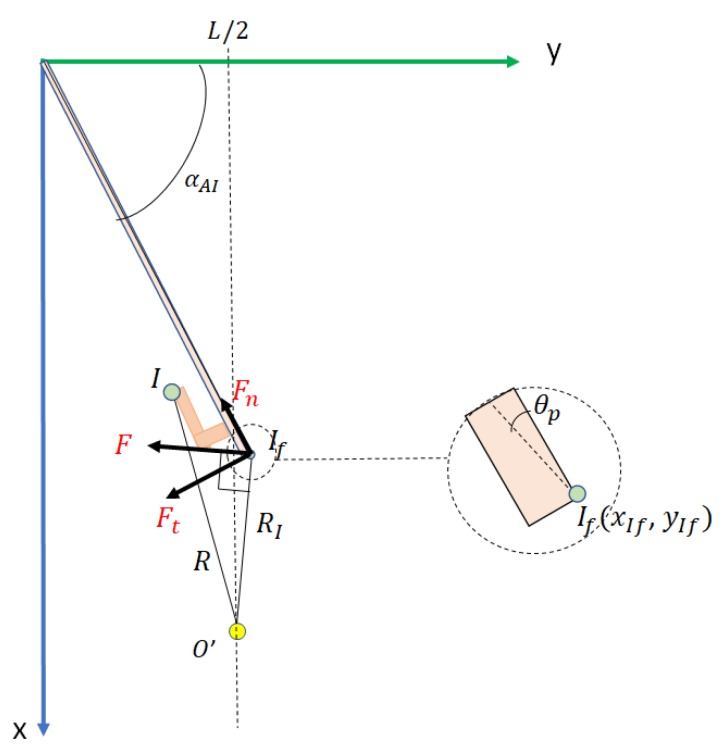
Forces undergone by the door edge at the contact point $I_{f}$ in phase 5.

When the current position of the robot in $O^{\prime }\left(x_{O}^{\prime },y_{O}^{\prime }\right)$ and the contact point $I_{f}\left(x_{If},y_{If}\right)$, the kinematics of the door pushing movement is performed in the same way as in Phase 3, but with several differences (see Figure 15):

The distance between $O^{\prime }$ and $I_{f}$, in the frame $S_{d}$, remains constant while the robot moves forward in the *X* direction. This distance $R_{If}$ is

(29) $$\begin{array}{c} R_{If}=\sqrt{{\left(x_{If}-x_{o}^{\prime }\right)}^{2}+{\left(y_{If}-y_{o}^{\prime }\right)}^{2}.} \end{array}$$

The end-effector makes contact with the door, but does not hold it. As a consequence, a pushing force *F* is exerted perpendicular to $\vec{O^{\prime }I_{f}}$. This decomposes into a tangential force $F_{t}$, which causes the door to rotate, and a normal force $F_{n}$, which is compensated by the rigidity of the door.

This phase ends when the door opening angle α is close to π/2. Note that if $v_{r}$ is constant, the angular velocity is now less than in phase 3:

(30) $$\begin{array}{c} w_{I}=\frac{v_{r}}{L}. \end{array}$$

The door opening angle is calculated as:

(31) $$\begin{array}{c} \alpha_{AI}=\alpha_{A}+\alpha_{I}, \end{array}$$

with $\alpha_{A}=w_{A}t_{A}$ and $\alpha_{I}=w_{I}t_{I}$, where $t_{I}$ is the time that has elapsed in this phase.

The coordinates of $I^{\prime }$ during its rotation movement are:

(32) $$\begin{array}{c} x_{If}^{\prime }=Lsin\left(\alpha_{AI}-\theta_{p}\right), \end{array}$$

(33) $$\begin{array}{c} y_{If}^{\prime }=Lcos\left(\alpha_{AI}-\theta_{p}\right), \end{array}$$

where $\theta_{p}=arctan\left(\frac{e}{L}\right)$ and *e* is the width of the door, and the coordinates of the base of the robot are:

(34) $$\begin{array}{c} x_{O}^{\prime }=\sqrt{R_{I}^{2}-{\left(y_{O}^{\prime }-y_{If}^{\prime }\right)}^{2}+x_{If}^{\prime }}, \end{array}$$

(35) $$\begin{array}{c} y_{O}^{\prime }=\frac{L}{2}. \end{array}$$

The coordinates of the end-effector with respect to the robot system $\left\{S_{O}\right\}$ are in Equations (22)–(24), now for $\left(x_{If}^{\prime },y_{If}^{\prime }\right)$.

### 5.6. Phase 6. Door Traversal

The manipulator moves to its home position. The robot then goes straight in coordinate *X* by a distance of $x_{O}^{\prime }$ and leaves the room, reaching the first scanning position of the new room.

## 6. Experimental Results

### 6.1. Open Doors

Real experimentation was performed with MoPAD on building floors with multiple rooms. Since the dimensions of the robot are 65×103×150 cm, the scenarios tested were composed of wide corridors and doors. Additionally, in order to ensure that MoPAD can traverse doors safely, it was necessary to take into account a safe robot distance of 30 cm. In summary, we experimented with 120 cm wide doors.

**Figure 16:**
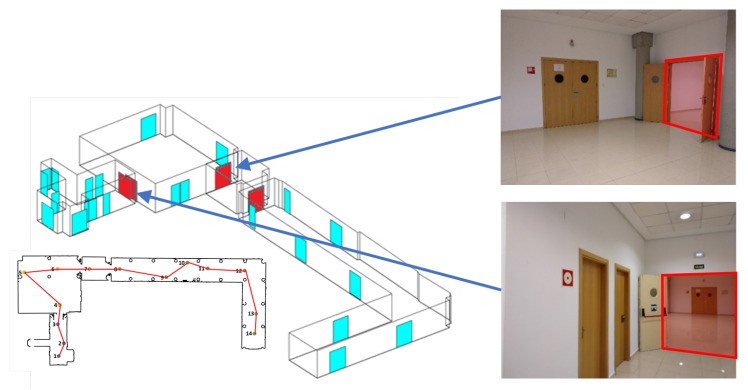
(Left) B-rep model generated by our system with traversed doors in red. The blueprint of the scene and the trajectory followed by MoPAD is shown below. (Right) Pictures taken from MoPAD and the recognized open doors highlighted in red.

A case study is shown in Figure 16. We show the results obtained in a part of the basement of the Industrial Engineering School building at Castilla La Mancha University (Spain). This scenario, of 19.5×44 m in size, is composed of 4 rooms and 32 doors, of which only three were open doors. The left-hand side of Figure 16 shows the B-rep model of the building extracted from the automatic digitization process. Open doors have been plotted in red and closed doors in blue. The blueprint of the scene, with the trajectory followed by MoPAD (in red), is presented below. Some pictures of the scene, with the open doors highlighted, are shown on the right.

**Figure 17:**
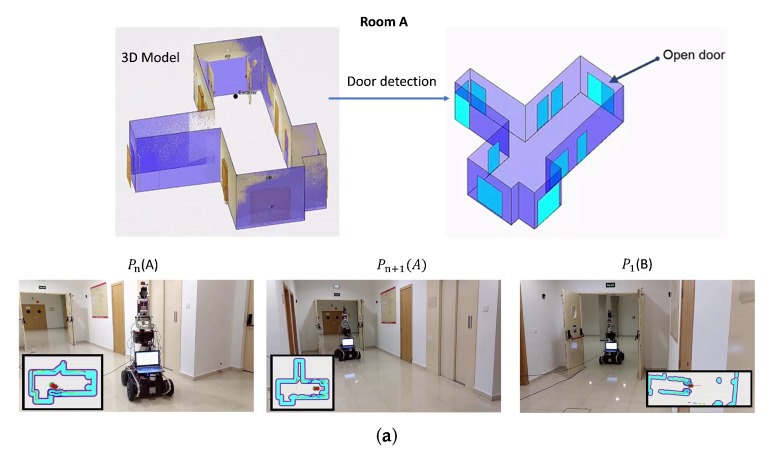

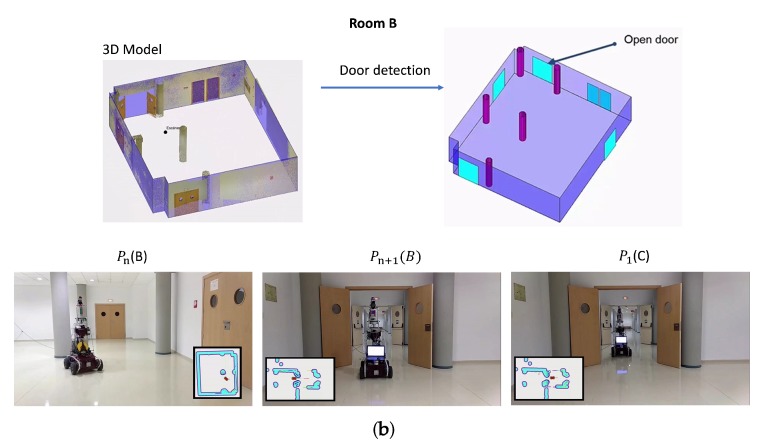
Open door traversal experimentation with MoPAD. (**a**) Open door traversal in room A. (Top) 3D model obtained by MoPAD after finishing the scanning process and detection of an open door. (Bottom) Pictures of MoPAD before and after going through the door. (**b**) Open door traversal in room B.

Figure 17a,b show more details of the results in two adjacent rooms (denominated as A and B). Once room A has been digitized, a 3D model containing the structural components of the room is generated. The door detection algorithm then locates the exit door (see Figure 17a, up). MoPAD then moves towards the door, leaves room A and enters room B. The bottom of Figure 17a shows MoPAD in different scan positions. In these figures, $P_{n}\left(A\right)$ represents the last scanning position in room A, $P_{n+1}\left(A\right)$ is the position before going through the door and $P_{1}\left(B\right)$ is the first scanning position of room B.

### 6.2. Closed Doors

Experimentation in the case of closed doors was carried out on simulated scenarios with the help of kinematic and dynamic simulation tools. First, the kinematic of the robot arm and the mobile robot were calculated using Matlab R2019a. This programming environment was also used to model the manipulator robot arm and the scene, which contains a closed door with a simple handle. Each of the aforementioned stages (Section 5) were run, providing the corresponding MoPAD’s and robot-arm’s trajectories.

**Figure 18:**
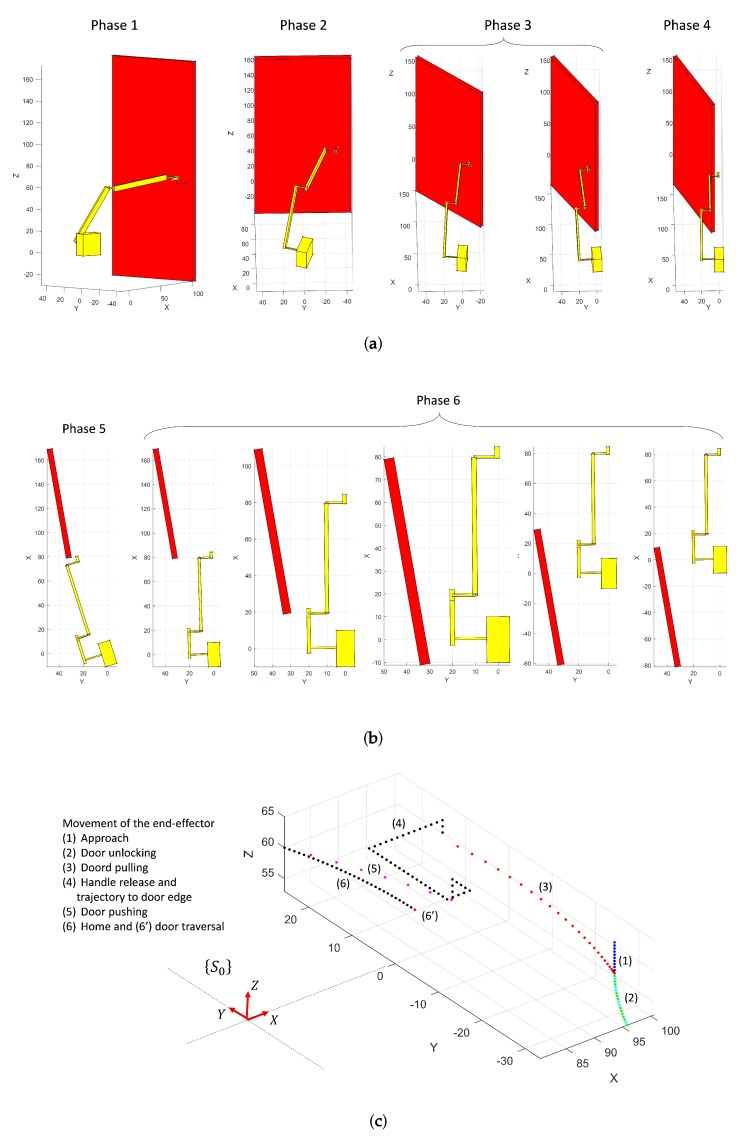
(**a**,**b**) Visualization of the robot arm and the door at different stages of the process. (**c**) Trajectory followed by the end effector throughout the entire process. Each stage is represented with a different colour.

**Figure 19:**
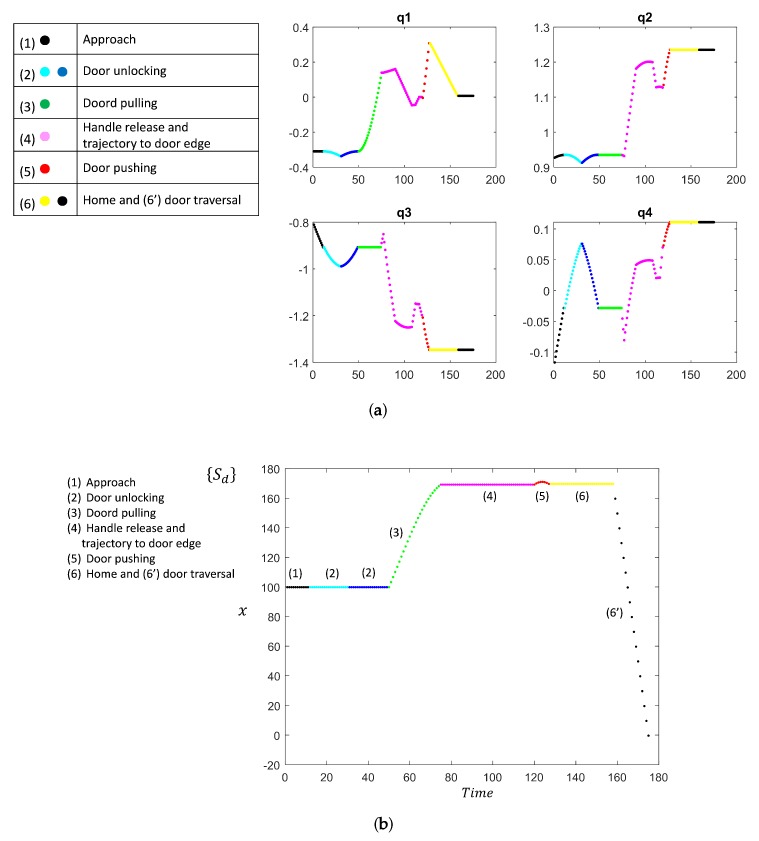
(**a**) Joint trajectories for different stages (in colours) in the door-opening process. Each stage is represented with a different colour. (**b**) MoPAD trajectory.

A visual representation is illustrated in Figure 18a,b. It shows shots of the robot arm interacting with the door in different stages of the door opening process. Figure 19 shows the joint trajectories of the robot arm and the trajectory followed by MoPAD.

The dynamic simulation was carried out using Gazebo, a widely used physics simulator that allows the customization of the parameters concerning the physical properties of each of the different components of the scene, such as those related to collisions or the elastic constant of the rotation of the door handle. Gazebo provides the possibility of simulating different robots, in a precise and efficient way, in complex indoor and outdoor scenarios.

**Figure 20:**
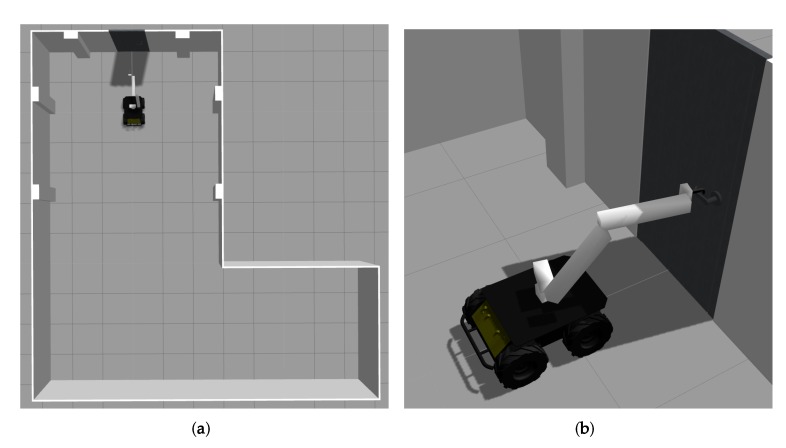
(**a**) Top view of the simulated environment. (**b**) Robotic platform interacting with the door handle.

Figure 20 shows the scene in Gazebo. MoPAD is simulated using the kinematics obtained from Matlab and by adding the dynamic component. The robot first approaches the door, interacts with the handle, opens the door and then traverses it. The complexity of this simulation tool means that chaotic and unexpected reactions sometimes occur, such as collisions of tremendous force between the final effector of the robot arm and the door handle. The reasons for these unstable reactions have yet to be established. As a result of this, the dynamic simulations performed are sometimes unstable and not very precise. It was, therefore, necessary to skip the stage in which the robot unlocks the bolt on the handle. Nevertheless, in the video attached in the supplementary material, the robot performs the remaining stages without any problems. Improving the quality of the simulations would be a top priority in any future improvements.

## 7. Conclusions and Future Work

The subject of passing through closed doors has been studied for many years and has been dealt with extensively as regards mobile robots in multiple environments and applications. However, this discipline is rarely addressed by researchers in the case of autonomous scanning platforms whose purpose is to digitize buildings. To date, the existing autonomous platforms are limited to scanning and navigating in indoor environments composed of several rooms, which are connected by open doors.

This work extends those earlier works to the case of floors of buildings in which some of the doors are closed. We present the robot–door interaction not only from a merely robotic point of view, but also from that of a complementary 3D data processing problem. The fact that the 3D scanner provides rich and valuable information of the scene makes the stages prior to this interaction more reliable and robust. Processes such as the detection of the door in the scene, the classification of the door type, the recognition/positioning of the door handle and the extraction of the rotation axis and the interaction point are, therefore, essential aspects in the whole solution.

In this paper, a simple 4 DOF robot-arm on board our MoPAD platform is presented as a valid alternative by which to carry out the opening process in several steps. This solution has been tested and evaluated by using Gazebo, a powerful simulation tool in which we can insert the real scanned geometry and simulate not only the kinematic of the robot arm, but also its dynamic behaviour and the existing collisions.

Having defined the formulation of the robot arm-door handle interaction and evaluated our solution using a simulated tool, the next step in our research will be its real implementation. A robot arm similar to that presented in this article will, therefore, be integrated into MoPAD in the near future.

## Figures and Tables

**Table 1:** Denavit–Hartenberg parameters of the manipulator.

Link	$l_{i}$	α	$d_{i}$	$q_{i}$
**1**	0	π/2	$d_{1}$	$q_{1}$
**2**	$l_{2}$	0	$d_{2}$	$q_{2}$
**3**	$l_{3}$	0	$d_{3}$	$q_{3}$
**4**	$l_{4}$	0	$d_{4}$	$q_{4}$

## Supplementary Materials

Supplementary File 1
